# The Patient Health Engagement Model in Cancer Management: Effect of Physical Activity, Distress Management, and Social Support Intervention to Improve the Quality of Life in Breast Cancer Patients

**DOI:** 10.1155/2022/1944852

**Published:** 2022-04-30

**Authors:** Maryam Changizi, Leila Ghahremani, Niloofar Ahmadloo, Mohammad Hossein Kaveh

**Affiliations:** ^1^Department of Health Education & Promotion, School of Health, Shiraz university of medical science, Shiraz, Iran; ^2^Department of Radiation Oncology, Namazi Hospital, Shiraz University of Medical Sciences, Shiraz, Iran

## Abstract

**Background:**

Providing better care and encouraging behaviors promoting health are effective measures to manage breast cancer patients. The present research was conducted to carry out an intervention based on the PHE model to promote physical activity, distress management, social support, and quality of life in breast cancer patients.

**Methods:**

This randomized control trial study was conducted on 123 women with breast cancer and their caregivers (group 1 = 61 and group 2 = 62). Subjects entered the study from the oncology ward of a specialized clinic in Shiraz, Iran. The education was done through clips, pamphlets, and lectures in 8 sessions with a duration of 15 to 25 minutes. A posttest was carried out in the both groups in 2 times (the end and 3 months) after intervention. The SPSS_**25**_ software was used to analyze the data.

**Results:**

The mean and SD of age of group 1 were (45.77 ± 8.84) and control group 2 (45.58 ± 77.64). The fiding showed a significant difference between the mean scores of distress management, social support, physical activity, and cancer self-efficacy in group 1 vs. group 2 after intervention (*P* < 0.001). Also, the educational intervention improved quality of life (*P* = 0.003).

**Conclusion:**

Intervention program based on the PHE model may facilitate the cognitive, emotional, and behavioral processes in breast cancer patient. Thus, it appears that the PHE model might promote patients' quality of life.

## 1. Introduction

**Figure 1:**
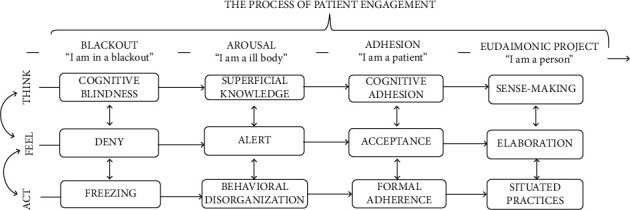
The Patient Health Engagement Model (PHE). Serena Barello, Guendalina Graffigna 2015.

Breast cancer is increasing in developing countries and other parts of the world [1]. Evidence indicates that over two-thirds of cancer patients are not active enough [2]. Being diagnosed with cancer results in the patient going through some mental distress over a period due to the threatening nature of this disease that could influence the patient's health behavior and decision-making [3]. Studies show that interventions could improve the mental distress experienced in cancer patients [4, 5]. In addition, Asian women who have breast cancer had a poor quality of life, and it seems necessary to support them [6]. Social support is an important aspect of modern cancer care. Some studies showed a direct relationship between social support and empowerment to battle breast cancer [7, 8]. Breast cancer patient must manage their chronic condition [8]. Besides, the diagnosis of a serious disease might disturb patients' ability to evaluate their cognitive and perceptual functions while influencing their emotional capacities for participation in the care and treatment process [9, 10]. Therefore, there is a need for a coherent theoretical structure that firstly applies only to chronic patients. Secondly, model focuses simultaneously on cognitive, emotional, and behavioral processes. Third, it recommends the appropriate skills to move from one phase to another. The PHE model (Figure 1) recently designed for this purpose. This model has defined patient engagement in emotional, cognitive, and behavioral dimensions following their health condition [10]. Patient engagement is a process indicating four practical situations including blackout, arousal, adhesion, and eudemonic project [10]. At the “blackout” phase, the patient has not adopted effective coping strategies to manage life opportunities, and patients tend to feel helpless at this stage [11]. At the “arousal” phase, patients pay attention to any clinical signal sent by their body and consider them “warnings” which makes them worried. At the “adhesion” phase, patients develop a better psychological acceptance of “being a patient” and their medical services [11]. At the “eudemonic project,” patients have completely accepted their health condition and try to manage it. Besides, they have accepted and realized that “the role of a patient” is merely one of their roles in life [11]. Given the importance of this issue, the present study was conducted to carry out based on the PHE model to promote physical activity, distress management, self-efficacy, social support, and quality of life in women with breast cancer.

## 2. Methods

### 2.1. Sample Size

**Figure 2:**
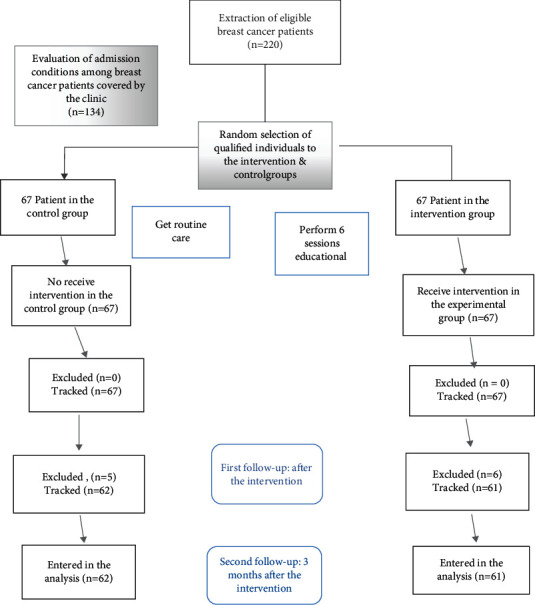
Study concert diagram.

The research is a randomized controlled trial. The research environment was the clinic's oncology ward in Namazi government educational and medical center of Shiraz in Iran 2020. the subjects were women with breast cancer and their caregivers. The sample size was estimated in accordance with previous study, *α* = 0.05 and *β* = 0.2, 134 people (67 people in each group) [12, 13]. First was studied 220 breast cancer patients referred to the oncology ward. Among them, 134 patients had inclusion criteria. Then, 134 caregivers also were appointed. However, 11 subjects were excluded from the study during the intervention. Finally, 123 people remained until the end of the study (group 1 = 61 and group 2 = 62), (Figure 2).

### 2.2. Sample Selection

Subjects were selected randomly. The names of the patients were extracted from the file in the oncology ward. After selecting patients to classify them into intervention and control groups, the block allocation method (4 blocks) was used. In each block, the first and third patients were in the intervention group, and the second and fourth patients were in the control group.

### 2.3. Inclusion Criteria

The inclusion criteria were as follows: breast cancer undergoing surgery, primary education, ability to participate in physical activity (no physical and motor limitations or orthopedic problems), age under 65 year, and patients in stage 2 based on the PHE model scale, having a smartphone.

### 2.4. Exclusion Criteria

The exclusion criteria were as follows: patient death, absence of more than two sessions in the intervention program, minimum literacy, and not having a smartphone.

### 2.5. Data Collection Tools

#### 2.5.1. Persian Version of the International IPAQ Physical Activity Questionnaire during the Last Seven Days

This questionnaire assesses physical activity in the last 7 days. It has 27 questions in 5 subscales called physical activity in leisure time (6 questions), domestic-related activities (6 questions), transportation (6 questions), work activity (7 questions), and time spent sitting (2 questions). Thus, the combination of moderate, intense physical activity, or walking for 7 days can be divided into three categories: low physical activity “METs-min/week” (less than 600 minutes per week), moderate activity (at least 600 minutes per week), and intense physical activity (at least 3000 minutes per week). This questionnaire's reliability and validity were reported in the study of Moghaddam et al. CVI = 0.85, CVR = 0.77, and Cronbach′s alpha = 0.7 [14].

#### 2.5.2. PHE Model Scale

This scale is the first tool dedicated to assessing the psychological experience engagement. We used the Persian version with acceptable psychometric properties. The PHE scale is an ordinal that considers four different positions. It has 5 items. This instrument assesses the degree of emotional elaboration reached by the patients concerning and their ability to engage in health [11]. The score for each question is 1-4. The validity of the original questionnaire version was reported to be 0.88 by the Pearson correlation test. In the present study, the validity of the questionnaire was first evaluated as a pilot and then in a cross-sectional study with a sample size of 129 women with cancer by factor analysis method and ordinal alpha = 0.626 was obtained.

### 2.6. Quality of Life Questionnaire

This is a cancer-specific measure of HRQOL. It includes 30 items to assess physical, role, emotional, cognitive and social functioning, global health, fatigue, pain, nausea and vomiting, dyspnea, insomnia, appetite loss, constipation, diarrhea, and financial problems with 4-point Likert scale (not at all, a little, quite a bit, very much) and score range (30-120). The EORTC QLQ-BR23 is for breasts and includes 23 questions to evaluate body image, sexual function, sexual pleasure, future prospects, side effects of systemic treatment, breast symptoms, arm symptoms, and discomfort of hair loss with 4-point Likert scale (not at all, a little, quite a bit, very much) and score range (23-92). The reliability (Cronbach's alpha) for all domains was reported 0.70 by Safaei et al. [15].

#### 2.6.1. Social Support Questionnaire

This instrument assesses perceived social support and has 16 items in 4 areas of family support (4 questions), friends (4 questions), spouse (4 questions), and others (4 questions), and it is scored in a 5-point Likert scale (strongly disagree to strongly agree, score range 16-80). Cronbach's alpha was reported for the Persian version in the study of Mirabzadeh et al. [16].

#### 2.6.2. Cancer Self-Efficacy Communication and Attitude Questionnaire

It has 19 questions that examine the self-efficacy of cancer patients. The questionnaire has a 6-point Likert scale (strongly disagree, moderately disagree, slightly disagree, slightly agree, relatively agree, strongly agree, score range 19-114) with 3 subscales including perception and participation in treatment, maintaining a positive attitude and seeking information. To assess the reliability, the test-retest method was used with a sample size of 50 pilots and paired *t*-test. For this questionnaire, CVR = 0.863 and CVI = 0.857 were obtained [17]. Cronbach's alpha was calculated 0.842 for this questionnaire.

#### 2.6.3. Distress Thermometer

This tool consists of 2 parts: one part including a checklist of practical, family, spiritual-religious, emotional, and physical problems with a yes-no scale (score range 0-38). The second part includes the distress thermometer, and score is between 0 and 10 (0 = no distress and 10 = extreme distress). The number 4 is the cutting point of this tool. A score 0-4 means no distress, a score of 4-7 means moderate levels of distress, and a score of 7-10 is severe distress. Its validity and reliability were reported in other studies [18].

### 2.7. Intervention

The intervention took 12 weeks and included seven training sessions as follows:

The first session is as follows: breast self-exam and social support education through the educational clip and text message (15 min). The second session is as follows: relaxation technique (muscle relaxation and guided image visualization) by sending pamphlet for distress management (10 min).

The third session is as follows: taught self-efficacy through the educational clip and text messages (15 min).

The fourth to the sixth sessions are as follows: text messages, video, and educational clip (30 min).

The seventh session is as follows: goal-setting skills by sending videos and text messages (15 min).

The eighth and ninth sessions are as follows: the social support and its importance for caregivers through the educational clip and text message (20 min).

### 2.8. Exercise Plan

Three weekly aerobic training sessions (warming up, main exercises, cooling down) took 35-40 minutes for the first and second weeks, 40-45 minutes for third and fourth weeks, and 45-55 minutes for the fifth week. Participants' questions were answered through phone calls during the intervention. Besides, the researcher created an online group in the Whatsapp application to teach physical exercises, review positive patient care experiences, and send daily messages to reinforce and support.

Data were collected in three time periods before the intervention immediately after and 3 months after the intervention. The data were analyzed with SPSS 26 software. We used descriptive, Mann–Whitney*U*, Friedman and Categorical Principal Component Analysis (CATPCA) tests.

## 3. Results

The mean and standard deviation of patients' age in the intervention and control groups were 45.78 ± 8.48 and 45.72 ± 7.60 years, respectively (*P* < 0.476). Most of the subjects were housewives in the intervention group and employees in the control group. The distribution of jobs in the two groups before the intervention was statistically significant (*P* < 0.001) see Table 1.

Anthropometric indices showed that more subjects in both groups were in the overweight range. The statistical test results did not show a significant difference between the intervention and control groups (*P* = 0.553). In the intervention group, subjects suffered from diabetes (11.5%), and a minority suffered from hypertension and thyroid disorders (4.2%). The control group suffered from thyroid disorders (11.3%), kidney and urinary tract disease (8.1%), diabetes 8.1%, and hypertension (6.5%). According to the checklist of the issues associated with distress, the most frequently reported physical problems and tingling are in the hands and legs (43.1%). In emotional problems, fear (24.4%), depression (8.9%), sadness (14.6%), anxiety (30.1%), and anger (20.3%) were reported. Based on the median and PHE scale, patients in the intervention group were placed in the third position of the model 3 months after the intervention, but no significant difference was seen in the control group(Table 2).

After the educational intervention, there was a significant difference in the mean scores of social support, self-efficacy, physical activity, distress, and quality of life in the intervention group compared to the control group (*P* < 0.001). The score of family and spouse social support in the intervention group before the intervention and three months after the intervention showed a difference (*P* < 0.001). The results showed that the mean scores of physical activity at work, domestic activity, self-efficacy subscale (seeking for information), subscale friends, and other social support before, after the intervention, and three months after the intervention in both groups did not show a significant difference (*P* = 0.05) Table 3.

The overall score of cancer-related quality of life before and three months after the intervention is different from the control group, and these changes are statistically significant (*P* < 0.001).

Most qualities of life dimensions changed significantly in the intervention group after the intervention, but cancer and arm symptoms and sexual and economic performance were not different from the control group after intervention (*P* = 0.05) Table 4.

## 4. Discussion

The present study investigated the effectiveness of an intervention based on the PHE model on initiating and promoting physical activity, distress management, and social support in breast cancer patients. Results of the present study indicate that the intervention improved the level of engagement and upgraded them into the third level (adhesion). In other words, increasing patients' self-confidence to control the disease must be a priority to transfer them from the arousal level to the adhesion level which requires support and encouragement to adapt to life and help achieve emotional balance at the emotional level [19]. It was reported that patients with low engagement levels in care improved after the intervention, which is similar to the present study [11]. The PHE model plays a vital role in patients' ability to play an active role in self-management and adherence to healthy behaviors [10]. some patients have low engagement in care and treatment. It seems that patient engagement can inform the patient and health provider about education and policies. In addition, patients feel their participation is important. Patient engagement may also enhance accountability and mutual understanding between patients and healthcare providers. [10, 20, 21]. In this regard, Graffigna et al. confirmed that patient engagement in health is an important factor in improving care quality [21]. Patients' engagement and families are equally important in all countries of the world. However, the priority of this concept and the way of doing it are very different now [20]. Our results indicated clear changes in self-efficacy scores after the intervention. Patients with higher self-efficacy are more psychologically compatible with the disease than those with low self-efficacy [22]. Self-efficacy appears to be an essential goal for health interventions to promote healthy behavior in cancer patients [23]. In the study of Rezaeian et al. after educational intervention, the self-efficacy of women with breast cancer undergoing radiation therapy in the intervention group increased significantly compared to the control group [22]. Change in the perception and participation in the treatment and positive attitude towards cancer (self-efficacy subscale) was seen in the intervention group. According to pundits, allowing patients to participate in caregiving situations will enhance caregiving and enable them to make informed and reciprocal participatory decisions in caregiving decisions [24]. Seeking information (self-efficacy subscale) did not change in both groups after the intervention. Most breast cancer patients received basic information about cancer at the time of diagnosis, and they were reluctant to read more. Knowing more about the disease seems to cause anxiety and fear for patients, and they prefer not to seek information [25]. In the present study, social support scores increased in the intervention group. This finding was similar to other studies. Spouse and family support was the most important component of social support [26, 27]. Evidence indicates that social support has a direct impact on cancer patients' health behavior [28]. In the present study, educating patient caregivers was to facilitate the increase of social support and physical activity.

In other words, the role of caregivers is evident in managing patients; so, they might play a role in both creating motivations for joining patients' treatment methods and facilitating the ability to do it [29]. According to present results, some breast cancer patients suffered from anxiety, depression, and distress. The mean scores of distress improved in the intervention group compared to the control group. Anthony et al. indicated relaxation is influential in reduction anxiety, depression, and distress in breast cancer patients [30]. In the present study, physical activity score was increased after intervention and consistent with the study of Mazloumi [31]. Also, cancer survivors who included physical activity in their daily routine reported positive physical recovery after completing cancer treatment [32]. In present study, no significant difference was seen in score of physical activity at work in the intervention group, because most of the patients in the intervention group were housewives. This result is similar to the findings of Jayamani et al. [33]. There is strong empirical evidence to support exercise performance as part of cancer care. However, the effect size of these outcomes has been reported to be small to moderate on average [34]. In the present research, cancer-related quality of life score in the intervention group is statistically significant different from the control group. Shobeiri et al. supports our findings [35]. It seems that physical activity intervention after breast cancer diagnosis may improve patients' quality of life, spirituality and psychological health, body image, and physical fitness. These results are similar to the studies of Mehnert and Dieli-Conwright [36, 37]. Some dimensions of quality of life after intervention did not show significant changes, and it is similar to Makluf et al. [38, 39]. Our result indicates that patient engagement might be a promising way in the field of healthcare education. Perhaps, patients' engagement in cognitive, emotional, and behavioral phases can play a role in disseminating information and training materials as a health ambassador. It is important to encourage people to ask questions or talk about their concerns. Engaging patient and their caregivers may help and encourage their understanding of health issues [40]. Some factors seem to potentially affect patient engagement that are related to patient demographic characteristics, health literacy, health conditions (disease severity), and social supports. Challenges(patients fear and passive role) can be overcome by improving communication and educating patients. However, in developing countries where resources are scarce, patient-family engagement may begin with educating and empowering individuals to identify their health needs and seek timely healthcare [41]. Patient-family engagement may begin with educating and empowering individuals to identify their health needs and seek timely healthcare [40]. However, patient and family engagement may begin with educating and empowering individuals to identify their health needs and seek timely healthcare [41]. One of the strength of present study is the use of PHE model designed for chronic patients exclusively. Very few studies have been performed based on this model. Besides, the present study has investigated several health behaviors that are quite important to improve breast cancer patients' health. This study's limitations coincided with the coronavirus pandemic, which took away the possibility of obtaining verbal and nonverbal feedback from the patients by our research team.

## 5. Conclusion

Overall, physical, mental function, and quality of life improved significantly after the intervention group's intervention. Physical activity is a nonmedical and unaggressive treatment with positive impacts on breast cancer patients to improve the quality of life of breast cancer patients. First, it is necessary to pay attention to their emotional state, strengthen self-efficacy in patients, and develop desired health behavior with social support and other skills. Patient health management (PHE) appears to be a reliable and scientific tool for navigating health intervention. However, this model has been designed for chronic patients exclusively. However, few studies have been conducted based on this model, and it is suggested that this model be used in research.

## Figures and Tables

**Table 1:** Demographic characteristics of patients in the intervention and control groups.

Variable		Group 1^∗^ *N* = 61		Group 2^∗∗^ *N* = 62		*P* value
		Percent	Frequency	Percent	Frequency	
Job	Self-employed	7	11.4	16	25.8	*P* < 0.001
	Retired	3	4.91	9	14.5	
	Employee	3	4.91	19	30.6	
	Housewife	51	83.60	18	29.5	

Education	Academic	17	27.9	16	25.8	*P* = 0.284
	Diploma	17	27.9	9	14.5	
	High school	15	24.6	19	30.6	
	Primary	12	19.7	18	29	

Marital status	Single	54	88.5	53	85.5	*P* = 0.114
	Married	7	11.4	9	14.5	

^∗^Group 1 = intervention,  ^∗∗^group 2 = control, Chi-square test.

**Table 2:** PHE scale score changes after the intervention in the group 1 and group 2.

Variable	Group 1^∗^					Group 2^∗∗^				
	Item 1	Item 2	Item 3	Item 4	Item 5	Item 1	Item 2	Item 3	Item 4	Item 5
After intervention	3	3	3	3	4	3	3	2	2	2

^∗^Group 1 = intervention, ^∗∗^group 2 = control, Descriptive test-median.

**Table 3:** Comparison of mean score of physical activity, self-efficacy, distress management, and social support in both groups before and after the intervention.

Variable	Group 1 (*n* = 61), group 2 (*n* = 62)							
	Physical activity		Self-efficacy		Distress management		Social support	
	Group 1^∗^	Group 2^∗∗^	Group 1	Group 2	Group 1	Group 2	Group 1	Group 2
Before intervention	1594 ± 108.1	1600 ± 966.6	84.5 ± 9.3	82.2 ± 8.7	3.6 ± 2.5	3.67 ± 2.92	52.4 ± 9.5	51.3 ± 8.7
After intervention	1950 ± 1031	1591.6 ± 956.7	97.2 ± 8.5	81.8 ± 8.2	2.4 ± 2.02	3.61 ± 2.87	56.7 ± 8.3	50.6 ± 8.8
3 months after	1950.3 ± 104.3	1591.9 ± 958.6	97.3 ± 8.1	81.7 ± 8.3	2.2 ± 1.8	3.51 ± 2.73	55.8 ± 7.7	50.5 ± 8.4
*P* value	*P* < 0.001	*P* = 0.974	*P* = 0.005	*P* = 0.161	*P* < 0.001	*P* = 0.675	*P* = 0.003	*P* = 0.350

^∗^Group 1 = intervention,  ^∗∗^group 2 = control, Friedman test.

**Table 4:** Comparison of quality of life score in intervention and control groups.

Variable	Group 1 (*n* = 61), group 2 (*n* = 62)					
	Before intervention		*P* value	3 months after intervention		*P* value
	Group 1	Group 2		Group 1	Group 2	
Total quality of life	193.2 ± 14.4	194.1 ± 13.1	< 0.001	203.01 ± 8.96	194.41 ± 12.2	0.675
Physical function	18.04 ± 2.22	18.08 ± 2.08	*P* = 0.136	18.37 ± 1.41	18.08 ± 2.08	0.043
Role performance	7.62 ± 0.859	7.56 ± 0.951	*P* = 0.722	7.92 ± 0.30	7.59 ± 0.93	0.01
Emotional function	14.44 ± 1.97	14.48 ± 1.96	0.908	15.19 ± 1.48	14.51 ± 1.80	0.024
Cognitive function	7.40 ± 0.901	7.25 ± 1.12	0.412	7.80 ± 0.60	7.43 ± 0.822	0.005
Social performance	7.52 ± 0.828	7.61 ± 0.732	0.532	7.85 ± 0.401	7.59 ± 0.734	0.018
Global health	6.01 ± 2.68	5.91 ± 2.66	0.846	7.2 ± 2.38	5.95 ± 2.60	0.005
Body image	13.18 ± 3.40	13.43 ± 3.88	0.634	14.44 ± 2.42	13.46 ± 2.78	0.04
Side effects of treatment	24.77 ± 4.34	25.04 ± 4.22	0.720	26.54 ± 2.49	25.17 ± 4.14	0.029
Future perspective	2.16 ± 4.34	2.91 ± 0.91	0.760	3.52 ± 0.622	2.93 ± 0.865	< 0.001
Breast symptom	15.54 ± 1.009	15.93 ± 0.30	< 0.001	15.59 ± 0.948	15.62 ± 0.853	0.842
Arm symptoms	11.06 ± 1.28	11.16 ± 1.25	0.889	11.09.81 ± 1.30	11.11 ± 1.22	0.884
Hair loss	3.62 ± 0.839	3.66 ± 0.767	0.106	3.96 ± 0.179	3.69 ± 0.737	0.781
Economic performance	3.04 ± 0.825	3.04 ± 0.818	0.908	3.09 ± 0.794	3.09 ± 0.824	0.742
Signs and symptoms of cancer	48.11 ± 4.66	48.32 ± 4.48	0.802	49.37 ± 3.2	48.37 ± 4.50	0.159
Sexual function	9.81 ± 1.96	9.85 ± 1.98	0.921	9.87 ± 2.003	10.29 ± 2.18	0.264

Group 1 = intervention, group 2 = control, Mann–Whitney *U* test.

## Data Availability

The data of the present research can be made available at the logical request of the relevant author.
